# Public health and epidemiology journals published in Brazil and other Portuguese speaking countries

**DOI:** 10.1186/1742-7622-5-18

**Published:** 2008-09-30

**Authors:** Mauricio L Barreto, Rita Barradas Barata

**Affiliations:** 1 Instituto de Saúde Coletiva, Federal University of Bahia, Salvador, Brazil; 2 Department of Social Medicine, School of Medical Sciences of Santa Casa de São Paulo, São Paulo, Brazil

## Abstract

It is well known that papers written in languages other than English have a great risk of being ignored simply because these languages are not accessible to the international scientific community. The objective of this paper is to facilitate the access to the public health and epidemiology literature available in Portuguese speaking countries. It was found that it is particularly concentrated in Brazil, with some few examples in Portugal and none in other Portuguese speaking countries. This literature is predominantly written in Portuguese, but also in other languages such as English or Spanish. The paper describes the several journals, as well as the bibliographic databases that index these journals and how to access them. Most journals provide open-access with direct links in the indexing databases. The importance of this scientific production for the development of epidemiology as a scientific discipline and as a basic discipline for public health practice is discussed. To marginalize these publications has implications for a more balanced knowledge and understanding of the health problems and their determinants at a world-wide level.

## Introduction

It is well known that papers written in languages other than English have a great risk of being ignored simply because these languages are not accessible to the international scientific community. This general observation must not be different regarding the epidemiological and public health scientific literature. The objective of this paper is to facilitate the access to the public health and epidemiology literature available in Portuguese speaking countries by present the journals, as well as the bibliographic databases that index these journals and how to access them. It was found that it is particularly concentrated in Brazil with some few examples from Portugal and none in other Portuguese speaking countries.

In Brazil, public health and epidemiological studies gained momentum in the 1970's, with the implementation of the first postgraduate programs in Public Health (or Collective Health, as it called in Brazil). By the end of the decade, the creation of the Brazilian Association of Collective Health (ABRASCO) and the constitution of its epidemiology commission played an important role in the strengthening of this area of knowledge. As of the 1990's, Brazilian Epidemiology Congresses held at triennial intervals opened room to promote the training of teachers and researchers, as well as of healthcare workers.

These facts had an impact on scientific production and, in the period from 1973 to 1992, Brazil already accounted for 60.7% of the papers on public health produced by researchers from Latin America, and published in indexed journals of the database ISI/Thomson Scientific [1]. This fact becomes more relevant when we note that with regard to clinical and biomedical research, the percentage of Brazilian production was 26.6% and 38.7%, respectively.

Currently, Brazil has 39 postgraduate programs in Collective Health, three of which are exclusively in Epidemiology and great part of them include epidemiology. The directory of research groups of the Brazilian National Research Council includes, as per the 2004 census, 506 groups with at least one line of research in epidemiology. The average composition of each group is 6.1 researchers [2].

Research funding in epidemiology is fundamentally raised from national agencies: National Research Council (CNPq), Agency for the Financing of Studies and Projects (FINEP), Department of Science and Technology of the Ministry of Health (DECIT/MS), and state-based research funding agencies in most of the country's 27 States. A small share of projects receives foreign funds from international funding agencies and bodies of the United Nations system. The past years recorded substantial increase of financial resources directed to health research [3].

## Bibliographic databases and Open Access

### LILACS-Latin American and Caribbean Literature in Health Sciences

LILACS database is produced in a cooperative manner by the institutions that integrate the *...Latin-American and Caribbean System of Information in Health Sciences*. LILACS registers scientific-technical literature in health produced by Latin American and Caribbean authors as of 1982 [4].

The main objectives of this database are bibliographic control and dissemination of Latin American and Caribbean technical and scientific literature in Health, not included in international databases. LILACS describes and indexes theses, books, book chapters, annals of congresses or conferences, technical-scientific reports, journal papers, and other publications related to Health [4].

Access to LILACS database can be obtained through a compact disc LILACS/CD-ROM and also at the *Virtual Health Library *[5] in the item *...Scientific Literature*, with links to sources of complementary information, particularly with a database of full versions of texts and online services that supplies document copies. LILACS/CD-ROM is issued every four months and may be purchased upon subscription by any user or library. LILACS database gathers up to 432,093 documents, 77.8% of which correspond to papers published in journals, 16.1% are monographs, 4.7% are thesis, and 1.4% constitutes what is called "grey literature", because it cannot be found easily through conventional channels such as publishers. Approximately 50% of the documents registered in the database are in Spanish and 40% in Portuguese. The rest corresponds to documents in English, French, Italian, and German. Brazilian productions account for 54% of documents and, of these, 68.5% were published inside the country. The remainder Brazilian production is distributed across different Latin American countries, particularly Argentina (4.4%), Mexico (2.2%), and Chile (1.9%) [4].

### SciELO – Scientific Electronic Library Online

SciELO [5] portal is a repository of scientific bibliography that provides free access to journal collections selected from six Latin American countries (Argentina, Brazil, Chile, Colombia, Cuba, and Venezuela) and two Iberian countries (Spain and Portugal). These collections total 450 titles in several areas of knowledge. The Public Health collection currently has 11 journals: *Cadernos de Saúde Pública*, *Ciência & Saúde Coletiva*, *Revista Brasileira de Epidemiologia *and *Revista de Saúde Pública*, *Revista de Saúde Pública da Colômbia*, *Revista Cubana de Saúde Pública*, *Salud Pública de México*, *Revista Española de Salud Pública*, *Gaceta Sanitária *(Spain), *Pan-American Journal of Public Health *(Pan American Health Organization), and *Bulletin of the World Health Organization *[5]. The first four are journals published in Brazil, SciELO, created in 1997 as the result of the cooperation between the Latin American and Caribbean Center of Information in Health Sciences and the São Paulo Research Supporting Foundation (FAPESP), has the objective of increasing and sustaining visibility, accessibility, quality, use, and impact of scientific journals. SciELO is one of the world's most important initiatives in free access and the most innovative and influential advance to strengthen journals from Brazil and other Latin America countries [5].

Between 2001 and 2006 SciELO received 795 requests for appraisal of journals to integrate its Brazil's open access collection. In this period, 572 titles were evaluated and 122 were (15.3%) approved to integrate the collection. SciELO uses strict quality criteria to select the journals that will comprise its open access database. Titles in Health account for 29% of Brazil's collection. SciELO is among the 10 most visited databases through Google Scholar and accounts for 14% of accesses to journals of the Directory of Open Access Journals (DOAJ). Collection papers can be accessed directly on the website [6] or through LILACS (Latin American Literature in Health Sciences), DOAJ, PubMed/MedLine, World Cat, WoS, Lattes platform CAPES' journal portal, and Google Scholar. The average cost of SciELO operation is US$ 5,800 per journal year, approximately US$ 114 per new paper included, US$ 14.55 per paper maintained on the database and 2 cents per full text download [7].

SciELO portal search system provides queries per journal, author, title, issue, type of research, and access to full text in *html *or *pdf*. For journals in its database, SciELO designs and makes available typical bibliometric information, such as impact factor, number of citations, number of accesses, and others.

The Public Health collection only comprises journals indexed in Medline. As of August 2007, the Managing Committee changed the rule, defining that Public Health journals entitled to be included in the collection should publish chiefly papers related to disciplines in the field of Public Health, including health politics, management, and planning, social sciences and public health, health promotion and interventions, and epidemiology.

## Epidemiology and public health Brazilian journals

In Brazil, there are approximately 839 scientific journals [8] that can be classified in the different fields of Health Sciences. In the SciELO database, there are 57 Brazilian journals on Health Sciences, being three in Tropical Medicine and eight in Public Health and Epidemiology. In many of the other journals papers of interest to Public health and Epidemiology are also eventually published (Table 1).

**Table 1:** Brazilian Health Sciences Journals in SciELO database, not classified as Public Health or Epidemiology

**Journals**	**Database**	**Language**
Arquivos de Neuro-Psiquiatria	ISI	English
Brazilian Dental Journal	ISI	English
Brazilian Journal of Medical and Biological Research	ISI	English
Memórias do Instituto Oswaldo Cruz	ISI	English
Revista Brasileira de Psiquiatria	ISI	Portuguese or English
Revista da Associação Médica Brasileira	ISI	Portuguese, English or Spanish
Revista da Sociedade Brasileira de Medicina Tropical	ISI	Portuguese or English
Revista do Instituto de Medicina Tropical de São Paulo	ISI	English
Anais da Academia Brasileira de Ciências	ISI	English
Acta Cirúrgica Brasileira	Medline	English
Arquivos Brasileiros de Cardiologia	Medline	Portuguese and English
Arquivos de Gastroenterologia	Medline	English
Brazilian Journal of Infectious Diseases	Medline	English
Brazilian Oral Research	Medline	English
Pesquisa Odontológica Brasileira	Medline	Portuguese
Pró-Fono Revista de Atualização Científica	Medline	Portuguese or English
Revista Brasileira de Otorrinolaringologia	Medline	Portuguese or English
Revista Odontologia da Universidade de São Paulo	Medline	Portuguese or English
Revista Dental Press de Ortodontia e Ortopedia Facial	Medline	Portuguese
Revista Latino-Americana de Enfermagem	Medline	Portuguese, and English or Spanish
São Paulo Medical Journal	Medline	English
Acta Ortopédica Brasileira	SciELO	Portuguese or English
Acta Paulista de Enfermagem	SciELO	Portuguese
Anais Brasileiros de Dermatologia	SciELO	Portuguese
Arquivos Brasileiros de Endocrinologia & Metabologia	SciELO	Portuguese
Arquivos Brasileiros de Oftalmologia	SciELO	Portuguese
Brazilian Journal of Cardiovascular Surgery	SciELO	Portuguese
Clinics	SciELO	English
International braz urol	SciELO	English
Jornal Brasileiro de Patologia e Medicina Laboratorial	SciELO	Portuguese
Jornal Brasileiro de Pneumologia	SciELO	Portuguese
Jornal Brasileiro de Psiquiatria	SciELO	Portuguese
Jornal de Pediatria	SciELO	Portuguese or English
Jornal de Pneumologia	SciELO	Portuguese
Jornal Vascular Brasileiro	SciELO	Portuguese
Journal of Applied Oral Science	SciELO	English
Journal of Epilepsy and Clinical Neurophysiology	SciELO	Portuguese or English
Radiologia Brasileira	SciELO	Portuguese or English
Revista Brasileira de Anestesiologia	SciELO	Portuguese or English
Revista Brasileira de Ciências Farmacêuticas	SciELO	Portuguese
Revista Brasileira de Coloproctologia	SciELO	Portuguese
Revista Brasileira de Educação Médica	SciELO	Portuguese
RevistA Brasileira de Farmacognosia	SciELO	English
Revista Brasileira de Fisioterapia	SciELO	Portuguese or english
Revista Brasileira de Ginecologia e Obsterícia	SciELO	Portuguese
Revista Brasileira de Hematologia e Hemoterapia	SciELO	Portuguese
Revista Brasileira de Medicina do Esporte	SciELO	Portuguese or english
Revista Brasileira de Reumatologia	SciELO	Portuguese
Revista CEFAC	SciELO	Portuguese
Revista da Escola de Enfermagem da USP	SciELO	Portuguese
Revista da Sociedade Brasileira de Fonoaudiologia	SciELO	Portuguese
Revista de Nutrição	SciELO	Portuguese or English
Revista de Psiquiatria Clínica	SciELO	Portuguese
Revista de Psiquiatria do Rio Grande do Sul	SciELO	Portuguese
Revista do Colégio Brasileiro de Cirurgiões	SciELO	Portuguese
Texto & Contexto-Enfermagem	SciELO	Portuguese

Two journals centered on epidemiology are regularly published in Brazil (*Revista Brasileira de Epidemiologia *and *Epidemiologia e Services de Saúde*) and 16 other journals on Collective Health, many of which with a large share of papers published on epidemiology. Of these 18 journals, 8 are in the SciELO database, one of which is on epidemiology. Table 2 presents the list of 18 journals on Epidemiology or Collective Health published in Brazil, indicating the indexation bases in which they are found, the language in which papers are published, and their frequency. All have a peer-review process to select the papers they publish. In general, funding for these journals is provided by Brazilian (National or State based) science and technology agencies.

**Table 2:** Leading Brazilian journals on public health and epidemiology and their indexing databases, 2007

Journal name	Bibliographic Database	First year available in electronic version	Language of publication	Frequency
Cadernos de Saúde Coletiva	LILACS	2002	Portuguese, English, Spanish or French	quarterly
Cadernos de Saúde Pública	JCR, Medline, SCOPUS, LILACS, Scielo Public Health, DOAJ	1985	Portuguese, Spanish or English	monthly
Ciência & Saúde Coletiva	Medline, SCOPUS, LILACS, Scielo Public Health, DOAJ.	1998	Portuguese, Spanish or English	bimonthly
Epidemiologia e Serviços de saúde	LILACS	2003	Portuguese	quarterly
História, Ciência, Saúde, Manguinhos.	Medline, LILACS, SCPOPUS Scielo Brazil collection, DOAJ.	1994	Portuguese, Spanish English or French	quarterly
Interface	Scielo Brazil collection, LILACS, SCOPUS, DOAJ.	2005	Portuguese, Spanish or English	semiannually
Physis	Scielo Brazil collection, LILACS, SCOPUS DOAJ.	2004	Portuguese, Spanish or English	semiannually
Revista Baiana de Saúde Pública	LILACS	2004	Portuguese	semiannually
Revista Brasileira de Epidemiologia	LILACS, SCOPUS Scielo Public Health, DOAJ.	1998	Portuguese, Spanish or English	quarterly
Revista Brasileira de Saúde Materno Infantil	LILACS, SCOPUS Scielo Brazil collection, DOAJ.	2002	Portuguese, Spanish or English	quarterly
Revista Brasileira de Vigilância Sanitária		2006	Portuguese, English or Spanish	quarterly
Revista Brasileira em Promoção da Saúde	Latindex and DOAJ	2003	Portuguese, English or Spanish	quarterly
Revista da Escola Mineira de Saúde Pública		2007	Portuguese, English or Spanish	bimonthly
Revista de Atenção Primária em Saúde		2003	Portuguese	semiannually
Revista de Saúde Pública	JCR, Medline, LILACS, SCOPUS, Scielo Public Health, DOAJ.	1967	Portuguese, Spanish or English*	bimonthly
Saúde e Sociedade	LILACS	1992	Portuguese, English, Spanish or French	every four months

Saúde em Debate	LILACS	Without electronic access	Portuguese	quarterly

With the exception of *Revista de Saúde Pública*, all journals publish texts in a single language, most typically Portuguese, followed by English. However, all papers have abstracts in English, in addition to Portuguese. A brief summary of the characteristics of each of these journals is provided in Appendix 1.

## Epidemiology and public health journals in Portugal

In Portugal SciELO database makes available for free access papers published by six journals on health sciences, none of them exclusively dedicated to the field of Public Health or specifically to epidemiology (Table 3). A brief summary of the characteristics of some of these journals is provided in Appendix 2.

**Table 3:** Portuguese Health Science Journals in SciELO database

**Journals**	**Database**	**Language**
Arquivos de Medicina	SciELO	Portuguese
Jornal Português de Gastrenterologia	SciELO	Portuguese
Psicologia, Saúde & Doenças	SciELO	Portuguese
Revista Portuguesa de Pneumologia	Medline	Portuguese
Revista Potuguesa de Ciências do Desporto	SciELO	Portuguese
Revista Portuguesa de Psicossomática	SciELO	Portuguese

## Indexation, performance and evaluation of journals

Table 4 presents some scientometric data for the journals in SciELO database [6]. The average number of citations per paper published in the period ranged between 0.52 for the journal *História, Ciência, Saúde *and 2.87 for the journal *Physis*. The average half-life of citations was more homogeneous, with values around three years. The length of seven years for the average half-life of citations to papers of the journal *Physis*, probably is a result of the predominance of theoretical essays in this publication. Although *Revista de Saúde Pública *publishes predominantly the results empirical research, it also has a length above the average getting to almost 5 years.

**Table 4:** Scientometric indicators of Brazilian journals on Public Health and Epidemiology in SciELO database, 1999 to 2007

Journal	Citation per paper	Mean life (a)	Citations self received (%)	Citations self granted (%)	Foreign authors (%)
Cadernos de Saúde Pública	1.78	3.92	**58.0**	6.6	6.2
Ciência & Saúde Coletiva	1.07	3.38	32.9	2.8	
História, Ciências, Saúde – Manguinhos	**0.52**	3.49	52.3	2.9	5.2
Interface	0.38	3.29	28.9	4.7	6.1
Physis	**2.87**	**7.04**	**3.7**	3.2	5.1
Revista Brasileira de Epidemiologia	0.77	3.33	19.2	**0.0**	**3.7**
Revista Brasileira de Saúde Materno Infantil	0.67	**2.61**	37.5	1.3	8.2

Revista de Saúde Pública	2.09	4.95	52.9	**8.7**	**9.2**

Another interesting piece of information presented in Table 4 is the percentage of citations received originating from papers published by the same journal. In this item, the two journals indexed in JCR are the ones that have the highest proportion of self-citations, corresponding to slightly more than half of the quotes in each journal. The low rate of self-citation of the journal *Physis *stands out. The proportion of self-citations granted is considerably lower; that is, out of the group of papers cited by the papers published in these journals, a small share corresponds to citations of papers published in the same journal. For any journal this number does not exceed 10 percent.

**Figure 1:**
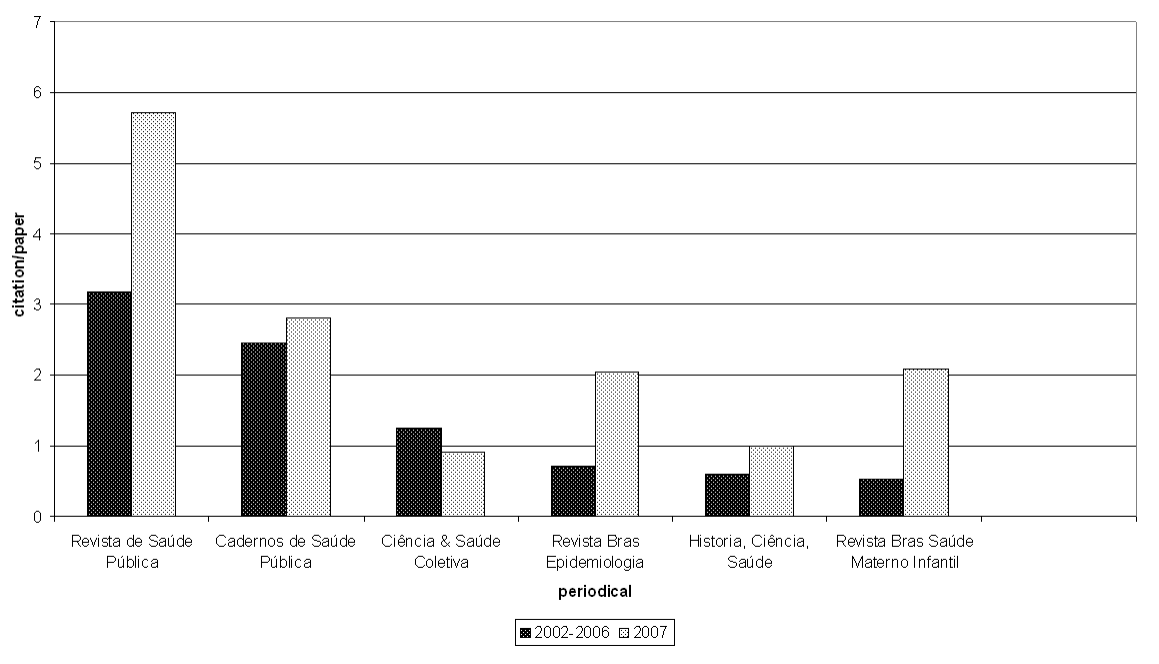
Citations per paper for journals of Public Health and Epidemiology, SciELO database 2002–2006 and 2007.

Figure 1 shows the average of citations per paper over the past five years until mid-2007. The number is quite higher than that in Table 4, showing that the trend in recent years has been to increase the average number of citations per paper published. The numbers for 2007 also exceed the average of the period for most journals.

Table 5 presents the local and foreign journals most often cited in the papers published by Brazilian Public Health and Epidemiology journals in SciELO database. The journals most often cited are those with the most visibility in their respective areas, showing the familiarity of Brazilian authors with global scientific production [6].

**Table 5:** National and foreign journals most cited in papers published in national journals of Public Health and Epidemiology, SciELO database, 1999–2007

Journal	Num. of citations	% citations granted
Revista de Saúde Pública	5600	30.0
Cadernos de Saúde Pública	4453	23.9
Lancet	1396	7.5
American Journal of Epidemiology	1119	6.0
Memórias do Instituto Oswaldo Cruz	1056	5.7
British Medical Journal	975	5.2
American Journal of Public Health	689	3.7
American Journal of Clinical Nutrition	586	3.1
Revista da Sociedade Brasileira de Medicina Tropical	521	2.8
Japanese Journal of Medical Science-Social Medicine	484	2.6
JAMA	451	2.4
Revista do Instituto de Medicina Tropical	392	2.1
American Journal of Tropical Medicine and Hygiene	369	2.0
Boletin de la Oficina Sanitaria Panamericana	298	1.6
Ciencia & Saúde Coletiva	273	1.5

TOTAL	18662	100.0

The monthly average downloads to full texts is around 7.5 million for all Brazilian journals in the SciELO collection. Over the past 5 years, three Public Health journals were among the most frequently visited: *Cadernos de Saúde Pública *(11,049,923 downloads), *Revista de Saúde Pública *(10,723,286 downloads), and *Ciência & Saúde Coletiva *(4,148,533 downloads) in the first, second and eleventh position, respectively [9].

*Cadernos de Saúde Pública *and *Revista de Saúde Pública *have also been among the most often cited journals of the database: *Cadernos de Saúde Pública *with 4,079 citations and *Revista de Saúde Pública *with 4,061 citations, were in the fourth and fifth positions, respectively [9].

Table 6 presents some data pertaining to statistics produced by SciELO, relative to eight selected journals. Even though each of them was included in the database at different times, it is possible to see that all of them are very frequently accessed with downloads of full papers. The number of downloads of the most accessed paper in each of them ranged between 300 and 27,900. A large number of papers received more than 500 downloads in each journal and the number of papers downloaded in full version was expressive in all journals, varying mainly due to the time of inclusion of the Journal in the database [5].

**Table 6:** Statistics of downloads of full paper in journals of Public Health and Epidemiology in SciELO database, 1999 to 2007.

Journal	Num. of downloads for paper most accessed	Num. of papers with more than 500 downloads	Year of inclusion in SciELO	Num. of papers downloaded
Cadernos de Saúde Pública	20981	1746	1999	3905460
Ciência & Saúde Coletiva	561	3	2002	26718
História, Ciência, Saúde, Manguinhos	22963	690		
Interface	9588	111	2005	253240
Physis	17745	71	2005	147659
Revista Brasileira de Epidemiologia	300	69	2005	10054
Revista Brasileira de Saúde Materno Infantil	27901	254	2003	805091

Revista de Saúde Pública	21667	1562	1999	3302642

The proportion of papers by Brazilian researchers with collaboration of foreign authors published in Brazilian journals is still relatively small. Between 5% and 9% of the papers have foreign researchers as co-authors. Parker and Meneghini [10] analyzed the papers with Brazilian authors in the database of Thomson Institute (ISI) in the period 1994–2003, in all areas of knowledge, that received more than 100 citations. They found that in those papers, international cooperation is as high as 84.3%. Limiting the analysis to the papers that received 250 citations or more, collaboration with foreign authors was observed in 89.2% of the papers [11].

## The Internet and Open Access

Teachers, researchers, students and employees of 188 higher education and research institutions throughout Brazil have immediate access to a considerable part of global scientific production updated through CAPES' journal portal, maintained by the government of Brazil at the annual cost of US$ 20 million.

CAPES' journal portal [9] provides free access to full versions of papers of more than 11,419 international, national and foreign journals, and to more than 90 databases with abstracts of documents in all areas of knowledge. It also includes a selection of important sources of academic information with free Internet access. The use of the portal is free for the users from participating institutions, that is, all public, philanthropic or for-profit universities whose graduate programs are credentialed through the three-yearly evaluation of their post-graduation courses. Access is provided through any terminal connected to the Internet located in the institutions or authorized by them [9].

All graduate, research and undergraduate programs of the Country gain in quality, productivity and competitiveness by using the Portal that is in constant development. Between January and June 2007, the number of accesses with request of full versions in the Portal was 7.4 million, which means an average cost of US$ 2.50 per paper.

## Discussion

Although English has been increasingly regarded as the *lingua franca *to communicate in sciences, Brazilian researchers, as it usually happens with researchers of other nationalities whose mother tongue is not English, normally face the dilemma between publishing their work in English, trying to increase their visibility in the international scientific community or publish in the native language seeking to have greater social impact, that is, using the results of their research to solve real local problems. This dilemma is very present in professional and applied areas such as Public Health [12].

Parker [13], analyzing the indexation structure of Latin American and Caribbean health science journals noted that there are about 1,000 journals in this area in the different countries. Of these, only 690 are indexed in LILACS database, whereas this number goes down to 141 for SciELO's portal. Only 65 can be found in Medline and just 28 in ISI-JCR. In this manner, it is quite clear that looking up less inclusive databases may lead to missing a significant part of the production. Considering that the indexation criteria for journals in LILACS database follow the same standards required by Medline, it is possible to conclude that 90% of the Latin American and Caribbean scientific production does not circulate in the international scientific community, probably due to the language barrier.

Clark and Castro [14] verified the impact that a search in LILACS database could have on the choice of papers for systematic reviews published in five medical journals with the highest impact factor. In 70% of the cases analyzed (62 systematic reviews), the search in LILACS identified new papers that could have been included and which had not been identified by authors.

Another important aspect that results in the exclusion of the literature published in languages other than English is emphasized by Freitas and collaborators [15]. The authors show that most of the clinical essays published in English show statistically significant results, while less than half of the studies published in other languages present positive results. The exclusion of the literature in Portuguese, Spanish and other languages may lead to significant biases in systematic reviews.

A systematic review about maternal morbidity and mortality searched scientific papers in eleven different databases, and about 20% of the papers included were published in languages other than English [16]. The authors concluded that for this type of systematic review, based on observational studies on the prevalence or incidence of a given health event, the search for evidence in regional databases that index the production of local journals not indexed in Medline is especially relevant.

Previous studies have documented that papers published in languages other than English have a great risk of being ignored, simply because they were written in languages that are not accessible to the international scientific community. However, "authors usually want to attract interest to their work both domestically and internationally" as Meneghini and Packer said [12]. According to these authors, Brazilian researchers publish about 50,000 papers every year, 60% of which are in Portuguese and 40% in other languages, mainly English. About 18,000 papers are published in journals indexed by the Thomson Scientific Web of Science database, 97.3% of them published in English.

Barreto [1] analyzed the Brazilian production in epidemiology indexed in MedLine between 1984 and 2004. Using a Boolean combination of many terms to select papers in epidemiology, in the early phase of the period, 1984–1989, it was possible to retrieve only 91 papers by Brazilian authors from this database accounting for 0.5% of the world production. Using the same search criteria, in the late phase of the period, 2000–2004, 1096 papers could be found, accounting for 1.1% of the world production. A significant share of these papers – 38% – was published in national journals and others in English, mainly in American (23%) or British (18%) journals. Of the papers published in domestic journals, 33% were in English. Of all epidemiology papers written by Brazilian authors in journals indexed in MedLine, 71% were written in English.

While most Brazilian Epidemiology and Public Health journals accept papers in Spanish and English, in addition to Portuguese, *Revista de Saúde Pública *has recently adopted the alternative of continuing to publish its papers, in Portuguese Spanish or English in its printed version, but to have for all of them originally published in Portuguese a translation into English in its electronic version. With this measure, the Journal intends to keep its social commitment with disseminating the Brazilian scientific production for healthcare workers in the country without failing to participate, as much as possible, in the international scientific debate.

In short, epidemiological research in Brazil is consolidating itself at the same speed as other scientific fields in the country. It seeks a higher degree of globalization, as it usually happens in other scientific arenas, but without neglecting its commitment with the social practice meant to expand the knowledge on the health situation and its determinants.

## Summary

1. In Brazil, scientific production in epidemiology has grown substantially in the past decade.

2. A considerable share of this production is being published in English; however, there is a considerable number of research results published only in Portuguese.

3. Brazil is the only Portuguese-speaking country that has a network of scientific journals in public health/epidemiology. As to the languages papers are published, these journals usually publish in Portuguese, English, or Spanish, although most of the papers are published in Portuguese.

4. The creation of the open-access database SciELO provided a decisive contribution to strengthen Brazilian public health journals, thus enhancing the communication of Brazilian epidemiologists with epidemiologists from the rest of Latin America, Spain, Portugal, and Portuguese-speaking African countries.

5. The indexation of the leading Brazilian journals in MedLine has provided a greater degree of globalization of the epidemiological research produced in the country.

6. The social commitment of the community of Brazilian epidemiologists with the improvement of the health of the Brazilian population and the development and enhancement of the National Health System is the cause for a substantial share of the production to continue to be published in Portuguese.

## Full text in Portuguese

The full text translation of this paper in Portuguese is provided as Additional File 1.

## Abstracts in alternative languages

The abstract of this editorial has been translated into the following languages by the following translators (names in brackets):

• Chinese – simplified characters (Mr. Isaac Chun-Hai Fung) [see Additional file 2]

• Chinese – traditional characters (Mr. Isaac Chun-Hai Fung) [see Additional file 3]

• French (Mr. Philip Harding-Esch) [see Additional file 4]

• Spanish (Ms. Annick Bórquez) [see Additional file 5]

## Competing interests

The authors declare that they have no competing interests.

## Authors' contributions

Both authors contributed equally for the study design, data collection, analysis and interpretation of the results. RB drafted the first version of the paper. MLB and RBB read and aproved the submited manuscript.

## Appendix 1 Summary of characterisitics of Epidemiology and public health journals published in Brazil

***Revista Brasileira de Epidemiologia ***is a quarterly publication edited by the Brazilian Association of Collective Health Postgraduate Programs – ABRASCO, since 1998, and aims at publishing original first-time published papers, with critical reviews on specific themes that can contribute to the knowledge and development of Epidemiology and related sciences.

***Epidemiologia e Serviços de Saúde ***is published by the Brazilian Ministry of Health. Its objective is to spread epidemiological knowledge to enhance the country's National Health System (SUS), as well as to communicate technical norms and resolutions of the Ministry of Health relative to disease control programs.

***Revista de Saúde Pública ***is the oldest of the group, created in 1967 by the School of Public Health of the University of São Paulo to replace the Archives of the School of Hygiene and Public Health, which had been published since 1947. Its mission is to publish and disseminate scientific work that is relevant to Public Health. Most papers published belong to the field of epidemiology. It is the only from this group of journal that has its impact factor measured by JCR – in 2006 it was 0.36. As of 2003, all texts have an electronic full version available in English. After selecting the paper on PubMed, the left upper corner will show the indication "*free full text available at SciELO.org English/Portuguese"*. This link goes straight to the paper's webpage in SciELO.

***Cadernos de Saúde Pública ***was created in 1985 by the National School of Public Health connected to Oswaldo Cruz Foundation, a research institution maintained by the Brazilian Ministry of Health. Its mission is to publish original papers that can contribute to the study of public health in general and related disciplines, such as epidemiology, nutrition, parasitology, ecology, and vector controls, environmental health, public policies, health planning, social sciences applied to health, and others.

***Ciência & Saúde Coletiva ***is one of the publications of the Brazilian Association of Collective Health Postgraduate Programs (ABRASCO), started in 1996. The journal is thematic and publishes debates, investigation results and analyses about specific themes considered relevant for Collective Health.

***História, Ciência, Saúde, Manguinhos ***is another publication of Oswaldo Cruz Foundation. It started in 1994 and is dedicated to documentation, research, and museology in sciences and health history. The journal publishes original articles and other relevant material.

***Interface – Comunicação, Saúde, Educação ***is a publication edited by São Paulo State University "Julio de Mesquita" (UNESP) and UNI Foundation (Laboratory of Education and Communication in Health, Department of Public Health, School of Medicine of Botucatu and Department of Education, Institute of Biosciences of Botucatu), directed to the integration of Health with Human Sciences, especially with Communication, Education, and university training. The journal Interface was launched in August 1997.

***Physis – Revista de Saúde Coletiva ***is a publication of the Institute of Social Medicine of the Rio of Janeiro State University, a public higher education institution funded by the State of Rio of Janeiro. *Physis *is a publication dedicated to production in the field of Collective Health, with emphasis on Human and Social Sciences, Health Policies, Planning and Management. Epidemiology themes are not frequently published in this journal. It started in 1991.

***Revista Brasileira de Saúde Materno Infantil ***is published by the Instituto Materno-Infantil of Pernambuco (IMIP) [17] and its mission is to release papers on biomedical, epidemiological, and socio-cultural aspects of women's and children's health. It is funded by the IMIP itself, and also by the Brazilian Ministry of Health and UNESCO.

***Cadernos de Saúde Coletiva ***is published by the Institute of Collective Health of the Federal University of Rio of Janeiro. It prints unpublished papers that are considered relevant to the area of public health. There is free access to the full version of papers through LILACS database or directly at the website [18].

***Revista Brasileira de Vigilância Sanitária ***is a technical-scientific publication whose purpose is to disseminate original and unpublished papers that may contribute to the knowledge and development of sanitary surveillance and related areas. It encompasses themes regarding services, products and technologies related to healthcare, evaluation of sanitary practices, sanitary surveillance programs and services, environmental health, worker's health, public policies, health planning, and others. It was created in 2006 and has not been indexed in any bibliographic database yet.

***Revista Brasileira em Promoção da Saúde ***is an official publication of the Health Sciences Center of the University of Fortaleza (UNIFOR). Its objective is to disseminate the scientific knowledge on health promotion to professionals from the areas of nutrition, nursing, pharmacy, physical therapy, speech therapy, occupational therapy, dentistry, physical education, medicine and other related areas. Papers can be freely accessed in the journal's website [19].

***Revista de Atenção Primária em Saúde ***is a publication of the Federal University of Juiz de Fora in partnership with the Brazilian Society of Family and Community Medicine and the Popular Education and Health Network. Among the journal's objectives, we may highlight building awareness among healthcare workers and authorities to foster and disseminate themes and research in primary healthcare. The journal has been published since 1997 and issues after 2003 are available for download at the website [20].

***Revista da Escola Mineira de Saúde Pública ***was created in 2007 as an outlet for the dissemination of the scientific production of healthcare workers. It is published by the School of Public Health of Minas Gerais' State Health Department. Issues can be accessed at the webpage [21]

***Saúde e Sociedade ***is the journal of São Paulo's Public Health Association. It edits unpublished articles of reflexive nature, research and knowledge updates, under the form of research and updating papers, analysis of major themes, theoretical, methodological and technical essays. It also publishes experience reports, biographies, interviews and testimonies. They value especially the papers that make an interface between health and human sciences. Papers can be freely accessed directly at the website [22].

***Revista Baiana de Saúde Pública ***was first published in March 1974. It is a regional journal and mainly publishes the results of studies and research in the field of public health produced in the Department of Health of the State of Bahia. The last volumes can be freely accessed at the website [23]

***Saúde em Debate ***is the journal of the Brazilian Center for Studies in Health (CEBES). It has been published since 1976 as a media for the discussion and dissemination of the Brazilian sanitary reform. It is not electronically available. It publishes mainly essays and position statements regarding political issues relevant to the improvement of the national healthcare system.

## Appendix 2: Summary of characterisitics of Epidemiology and public health journals published in Portugal

During the period spanning from 1996 to 2002, six issues of the ***Revista de Epidemiologia ***were published, which were in fact supplements of *Arquivos of Medicina*, recently included into SciELO Portugal. These six issues are available for free access at the website of the Portuguese Association of Epidemiology [24].

***Revista Portuguesa de Saúde Pública ***has been edited by the National School of Public Health, of the New University of Lisbon, since 1983. Its publication is semi-annual, and, as of 1999, it has also counted on the publication of theme-specific issues. The mission of the journal is to contribute to the production of scientific knowledge in the area of public health, promoting its discussion and development in Portugal and in the world. Its texts are published in Portuguese with an abstract in English. The issues published between 2000 and 2007 are available for free access and older issues will gradually be posted in electronic media. Access through the University's webpage is not really simple, however it is possible to have easy access through Google Scholar.

## Supplementary Material

Additional file 1 Full text article in Portuguese. Click here for file

Additional file 2 Abstract in simplified Chinese Click here for file

Additional file 3 Abstract in traditional Chinese. Click here for file

Additional file 4 Abstract in French. Click here for file

Additional file 5 Abstract in Spanish Click here for file
