# Supplementary material for: Public health and epidemiology journals published in Brazil and other Portuguese speaking countries
Source: Emerg Themes Epidemiol. 2008 Sep 30;5:18. doi: 10.1186/1742-7622-5-18 (PMC2572600; doi:10.1186/1742-7622-5-18)
Supplement: Additional file 1 — Full text article in Portuguese. [file 1742-7622-5-18-S1.doc]

**REVISTAS DE SAÚDE PÚBLICA E EPIDEMIOLOGIA PUBLICADOS NO BRASIL E EM OUTROS PAÍSES DE LINGUA PORTUGUESA**

Maurício L Barreto

Instituto de Saúde Coletiva, Universidade Federal da Bahia, Brasil

e-mail: maurício@ufba.br

Rita Barradas Barata

Departamento de Medicina Social, Faculdade de Ciências Médicas da Santa Casa de São Paulo

e-mail: rita.barata@fcmscsp.edu.br

As pesquisas epidemiológicas no Brasil ganharam grande impulso a partir da década de 70 com a implantação dos primeiros cursos de pós-graduação em Saúde Pública no país. A criação no final da década, da Associação Brasileira de Pós-graduação em Saúde Coletiva (ABRASCO) e a constituição de sua comissão de epidemiologia desempenharam importante papel no fortalecimento desta área de conhecimento. A realização, a partir de 1990, de congressos brasileiros de epidemiologia a intervalos trienais, propiciou espaço para a divulgação da produção tanto de docentes e pesquisadores quanto de profissionais de saúde com atuação na área.

Estes fatos tiveram repercussão na produção científica e no período de 1973-19992 o Brasil já era responsável por 60,7% dos artigos na área de saúde pública produzidos por pesquisadores da América Latina, e publicados em periódicos indexados da base bibliográfica do ISI/Thomson Scientific.^1^ Este dado torna-se mais importante quando observa-se que com relação à pesquisa clinica e à pesquisa biomédica básica a percentagem da produção brasileira era de 26.6% e 38.7%, respectivamente.

No momento existem no país 39 programas de pós-graduação em Saúde Coletiva, dos quais três são exclusivos em Epidemiologia. O diretório de grupos de pesquisa do Conselho Nacional de Pesquisas tem cadastrado, no censo de 2004, 506 grupos com pelo menos uma linha de pesquisa em epidemiologia. A composição média de cada grupo é de 6,1 pesquisadores. ^2^

O financiamento das pesquisas em epidemiologia é feito fundamentalmente através das agências de fomento do sistema nacional de ciência e tecnologia: Conselho Nacional de Pesquisa (CNPq), Financiadora de Estudos e Projetos FINEP), Departamento de Ciência e Tecnologia do Ministério da Saúde (DECIT/MS) e Fundações Amparo à Pesquisa existentes na maioria dos 27 Estados que formam o país. Pequena parcela dos projetos recebe financiamento externo de agências de fomento e órgãos do sistema das Nações Unidas. Nos últimos cinco anos observou-se aumento substancial dos recursos financeiros direcionados para a pesquisa na área da Saúde.^3^

**Bibliographic databases and Open Access**

LILACS- Literatura Latino-americana e do Caribe em Ciências da Saúde

A base de dados LILACS, é produzida de forma cooperativa pelas instituições que integram o [*Sistema Latino-Americano e do Caribe de Informação em Ciências da Saúde*](http://www.bireme.br/bvs/P/psystem.htm). A LILACS registra a literatura científico-técnica em saúde produzida por autores latino-americanos e do Caribe publicada a partir de 1982.^4^

Os principais objetivos desta base de dados são o controle bibliográfico e a disseminação da literatura científico-técnica latino-americana e do Caribe na área da Saúde, ausente das bases de dados internacionais. Na LILACS são descritos e indexados: teses, livros, capítulos de livros, anais de congressos ou conferências, relatórios técnico-científicos, artigos de revistas, etc., relacionados à área da Saúde.^4^

O acesso à base de dados LILACS pode ser realizado em disco compacto LILACS/CD-ROM e também integralmente na [*Biblioteca Virtual em Saúde*](http://www.bireme.br/) *(www.bvs.org.br)* no item [*Literatura Científica*](http://bases.bvs.br/public/scripts/php/page_show_main.php?home=true&lang=pt&form=simple), com conexões a fontes de informação complementares, particularmente com bases de dados de textos completos e serviços online de fornecimento de cópias de documentos.^4^

O LILACS/CD-ROM possui periodicidade quadrimestral e inclui as bases de dados do Sistema LILACS, bases de dados de Centros Especializados e da Biblioteca da Organização Pan-Americana da Saúde, e a base de dados SeCS - Seriados em Ciências da Saúde. Pode ser adquirido por assinatura por qualquer usuário ou biblioteca. A política de distribuição de LILACS/CD-ROM aos Centros Cooperantes é baseada na [contribuição](http://bvsmodelo.bvsalud.org/site/lilacs/P/contrib.htm) para a base de dados LILACS.^4^

A base LILACS reúne até 432.093 documentos dos quais 77,8% correspondem a artigos publicados em periódicos, 16,1% a monografias, 4,7% a teses e 1,4%, grande parte deste material constitui o que se denomina de "literatura cinzenta", pois cannot be found easily through conventional channels such as publishers. Cerca de 50% dos documentos registrados na base são escritos em espanhol e 40% em português. Os demais correspondem a documentos em inglês, francês, italiano e alemão. As produções brasileiras correspondem a 54% dos documentos e destas 68,5% foram publicadas no próprio país. O restante da produção brasileira distribui-se nos diferentes países latino-americanos predominando a Argentina (4,4%), o México (2,2%) e o Chile (1,9%). A produção brasileira é a que apresenta maior diversidade de locais de publicação.^4^

SciELO – Scientific Eletronic Library Online

O portal SciELO^5^ é um repositório de bibliografia científica que permite acesso livre a coleções de periódicos selecionados de 6 países latinoamericanos (Argentina, Brazil, Chile, Colômbia, Cuba, e Venezulea) e 2 ibéricos (Espanha e Portugal). Essas coleções totalizam 450 títulos em várias áreas do conhecimento. A coleção Saúde Pública conta atualmente com 11 periódicos: quatro periódicos brasileiros (Cadernos de Saúde Pública, Ciência & Saúde Coletiva, Revista Brasileira de Epidemiologia e Revista de Saúde Pública), a Revista de Saúde Pública da Colômbia, Revista Cubana de Saúde Pública, Salud Pública de México, Revista Española de Salud Pública, Gaceta Sanitária (Espanha), Panamerican Journal of Public Health (PAHO) e Bulletin of the World Health Organization.^5^

A criação do SciELO em 1997 possibilitada pela cooperação entre o Centro Latino-Americano e do Caribe de Informação em Ciências da Saúde e a Fundação de Amparo à Pesquisa do Estado de São Paulo (FAPESP), tem por objetivo aumentar e sustentar a visibilidade, acessibilidade, qualidade, uso e impacto de periódicos científicos. O SciELO representa uma das mais importantes iniciativas mundiais no movimento de acesso aberto e o avanço mais inovador e de maior impacto para o fortalecimento de periódicos brasileiros e dos demais países da América Latina.^5^

Entre 2001 e 2006, foram recebidos 795 pedidos de avaliação de periódicos para integração à base SciELO, coleção Brasil. Nesse período 572 títulos foram avaliados e 122 (15,3%) aprovados. Os títulos da área da Saúde representam 29% da coleção Brasil. O SciELO está entre as 10 bases mais acessadas através do Google Scholar e responde por 14% dos acessos a periódicos do Directory of Open Acess Journals (DOAJ).^6^ Os artigos das coleções podem ser acessados diretamente no site (www.scielo.br) ou através de links encontrados no LILACS (Literatura Latinoamericana em Ciências da Saúde), DOAJ, PubMed/MedLine, World Cat, WoS, plataforma Lattes, portal de periódicos CAPES e Google Scholar.

Os custos médios de operação do SciELO são de US$ 5,800 por periódico ano, cerca de US$ 114 por artigo novo incluído, US$ 14,55 por artigo mantido na base e 2 cents por download de texto completo.^6^

O sistema de buscas do portal SciELO permite a localização por periódicos, autores, títulos, assuntos, tipo de pesquisa e o acesso ao texto completo em htlm ou pdf. Para os periódicos contidos em sua base, o SciELO elabora e torna disponíveis as informações bibliométricas habituais tais como fator de impacto, número de citações, número de acessos e outras.

A coleção Saúde Pública era formada apenas por periódicos indexados no Medline. A partir de agosto de 2007 o comitê Gestor modificou esta regra, definindo que os periódicos de Saúde Pública passíveis de serem incluídos na coleção devem publicar majoritariamente artigos relacionados com disciplinas do campo da Saúde Pública, incluindo política, planejamento e gestão em saúde, ciências sociais e saúde pública, promoção e intervenções de saúde e epidemiologia; pertencer a uma coleção nacional certificada e demonstrar cumprimento da periodicidade de publicação.

**Epidemiology and Public Health Brazilian Journals,**

No Brasil existem em torno de 839 revistas científicas^7^ que podem ser classificadas nas Áreas das Ciências da Saúde. Na base SciELO – Scientific Eletronic Library Online (www.scielo.org), que utiliza critérios rígidos de qualidade para selecionar os periódicos que comporão a sua base de open-acess, existem 57 periódicos brasileiros na área de Ciências da Saúde dos quais 38 são revistas de biomedicina, sendo 3 em Medicina Tropical e 8 em Saúde Pública e Epidemiologia. Em muitos desses 38 periódicos, eventualmente poderão ser publicados artigos de interesse para a Saúde Pública e Epidemiologia. (anexo 1) .

São regularmente publicadas no Brasil duas revistas centradas em epidemiologia (Revista Brasileira de Epidemiologia e Epidemiologia e Serviços de Saúde) e 16 outras revistas na área da Saúde Coletiva, muitas delas com uma grande proporção dos artigos publicados na área da epidemiologia. Destas 18 revistas 8 estão na Base SCIELO, sendo uma de epidemiologia. A Tabela 1 apresenta a relação dos 18 periódicos em Epidemiologia ou Saúde Coletiva publicadas no Brasil, indicando as bases de indexação nas quais se encontram, a língua em que os artigos são publicados e a periodicidade. Todas tem processo de peer-review para selecionar os artigos que publicam.

Em geral o financiamento destas revistas é provido por recursos obtidos junto a agências de fomento do sistema público de ciência e tecnologia do país. Algumas destas agencias tem linhas especificas de apoio a publicações científicas.

Com exceção da Revista de Saúde Pública, em todas elas os textos estão disponíveis em uma única língua, em geral português, seguida pelo o inglês. Porém todos os artigos contam com resumos em inglês, alem do português. Em seguida é apresentado um breve sumario das características de cada uma destas revistas:

A **Revista Brasileira de Epidemiologia** é uma publicação trimestral, editada pela Associação Brasileira de Pós-Graduação em Saúde Coletiva - ABRASCO, desde 1998, e tem por finalidade publicar artigos originais e inéditos, inclusive de revisão crítica sobre temas específicos, que contribuam para o conhecimento e desenvolvimento da Epidemiologia e ciências afins.

A revista **Epidemiologia e Serviços de Saúde** é publicada pelo Ministério da Saúde do Brasil. Tem como objetivo a difusão do conhecimento epidemiológico para o aprimoramento do Sistema Nacional de Saúde do país (SUS), bem como normas técnicas e resoluções do Ministério da Saúde relativas aos programas de controle de doenças.

A **Revista de Saúde Pública** é a mais antiga do grupo, tendo sido criada em 1967 pela Faculdade de Saúde Pública da Universidade de São Paulo em substituição aos Arquivos da Faculdade de Higiene e Saúde Pública que vinham sendo publicados desde 1947. Sua missão é publicar e disseminar produtos do trabalho científico que sejam relevantes para a Saúde Pública. Grande parte dos artigos publicados pertence ao campo da epidemiologia. É a única das revistas deste grupo que tem seu fator de impacto mensurado pelo JCR, que em 2006 foi de 0.36. A partir de 2003 todos os textos têm versão eletrônica em inglês disponível na íntegra. No PubMed após selecionar o artigo aparecerá no canto superior esquerdo do resumo a indicação "*free full text avaliable at SciELO.org english/ portuguese"***.** Este link remete diretamente à página do artigo na SciELO.

O **Cadernos de Saúde Pública** foi criado em 1985 pela Escola Nacional de Saúde Pública vinculada à Fundação Oswaldo Cruz, instituição de pesquisa mantida pelo Ministério da Saúde do Brasil. . Tem por missão publicar artigos originais que contribuam para o estudo da saúde pública em geral e disciplinas afins, como epidemiologia, nutrição, parasitologia, ecologia e controles de vetores, saúde ambiental, políticas públicas e planejamento em saúde, ciências sociais aplicadas à saúde, dentre outras.

**Ciência & Saúde Coletiva** é uma das publicações da Associação Brasileira de Pós-graduação em Saúde Coletiva (ABRASCO), iniciada em 1996. A revista é temática e publica debates, análises e resultados de investigações sobre temas específicos considerados relevante para a Saúde Coletiva.

**História, Ciência, Saúde, Manguinhos** é outra publicação da Fundação Oswaldo Cruz, iniciada em 1994 e devoted to documentation, research, and museology in the history of the sciences and health. The journal publishes original articles and other material related to the history of the sciences and health.

A revista **Interface - Comunicação, Saúde, Educação** é uma publicação editada pela Fundação UNI e UNESP (Laboratório de Educação e Comunicação em Saúde, Departamento de Saúde Pública, Faculdade de Medicina de Botucatu e Departamento de Educação, Instituto de Biociências de Botucatu), voltada à articulação das Ciências da Saúde com as Humanidades, especialmente com a Comunicação, a Educação e a formação universitária. A Universidade Estadual Paulista "Julio de Mesquita" (UNESP) é uma das três universidades públicas mantidas pelo Governo do Estado de São Paulo. A revista Interface foi lançada em agosto de 1997.

**Physis – Revista de Saúde Coletiva** é uma publicação do Instituto de Medicina Social da Universidade do Estado do Rio de Janeiro, instituição pública de ensino superior custeada pelo Estado do Rio de Janeiro. Physis é uma publicação dedicada à production in the field of Collective Health, with emphasis in Human and Social Sciences, and Health Politics, Planning and Management. A publicação de temas em epidemiologia é pouco freqüente nesse periódico. Sua publicação iniciou-se em 1991.

A **Revista Brasileira de Saúde Materno Infantil** é publicada pelo Instituto Materno Infantil de Pernambuco ([IMIP](http://www.imip.org.br/)) e sua missão é a divulgação de artigos sobre os aspectos biomédicos, epidemiológicos e socioculturais da saúde da mulher e da criança. Ela é financiada pelo próprio IMIP e também pelo ministério da Saúde do Brasil e UNESCO.

**Cadernos de Saúde Coletiva** é editado pelo Instituto de Estudos em Saúde Coletiva da Universidade Federal do Rio de Janeiro e publica trabalhos considerados inéditos e relevantes para a área da saúde pública. Os artigos podem ser obtidos com texto completo em acesso livre através da base LILACS ou diretamente no site *www.nesc.ufrj.br/cadernos.*

A **Revista Brasileira de Vigilância Sanitária** é uma publicação de caráter técnico-científico que tem por finalidade divulgar artigos originais e inéditos que contribuam para o conhecimento e desenvolvimento da vigilância sanitária e áreas afins, abrangendo temáticas referentes a serviços, produtos e tecnologias relacionados à saúde, avaliação de práticas sanitárias, programas e serviços de vigilância sanitária, saúde ambiental, saúde do trabalhador, políticas públicas, planejamento em saúde, entre outros. Foi criada em 2006 não contando ainda com indexação em nenhuma base bibliográfica.

A **Revista Brasileira em Promoção da Saúde** é o órgão oficial do Centro de Ciências da Saúde da Universidade de Fortaleza (UNIFOR). Tem como objetivo divulgar o conhecimento científico sobre promoção da saúde para profissionais da área de nutrição, enfermagem, farmácia, fisioterapia, fonoaudiologia, terapia ocupacional, odontologia, educação física, medicina e demais interessados. Os artigos podem ser acessados livremente no site da revista.

A **Revista de Atenção Primária em Saúde** é uma publicação da Universidade Federal de JuizdeFora em parceria com a Sociedade Brasileira de Medicina de Família e Comunidade e da Rede de Educação Popular e Saúde. Entre os objetivos da revista podemos destacar o de sensibilizar profissionais e autoridades da área de saúde e estimular e divulgar temas e pesquisas em APS. A revista é publicada desde 1997 e os números a partir de 2003 estão disponíveis para *download* no *site www.nates.ufjf.br*.

A **Revista da Escola Mineira de Saúde Pública** foi criada em 2007 como veículo de divulgação da produção científica dos profissionais de saúde. Ela é editada pela Escola de Saúde Pública da Secretaria de Estado da Saúde de Minas Gerais. Os números podem ser acessados na página *www.esp.mg.gov.br/piblicacoes*.

A revista **Saúde e Sociedade** é a revista da Associação Paulista de Saúde Pública. Publica matéria inédita de natureza reflexiva, de pesquisa e atualização do conhecimento, sob a forma de: artigos de pesquisa e de atualização; análise de grandes temas; ensaios de natureza teórica metodológica e técnica. Publica, também relatos de experiência; biografias, entrevistas e depoimentos. São particularmente valorizados artigos que façam interface da saúde com a área de ciências humanas. Os artigos podem ser acessados diretamente do site em acesso livre.

**Revista Baiana de Saúde Pública**, criada em março de 1974, é uma revista de caráter regional e divulga principalmente resultados de estudos e pesquisas no campo da saúde pública, produzidos no Estado da Bahia.

A revista **Saúde em Debate** é a revista do Centro Brasileiro de Estudos em Saúde (CEBES), publicada desde 1976 como veículo de discussão e divulgação da reforma sanitária brasileira. Não está disponível em meio eletrônico. Publica principalmente ensaios e posicionamentos relativos às questões políticas relevantes para o aprimoramento do sistema único de saúde.

**Jornais Portugueses de Epidemiologia e Saúde Pública**

Na base SciELO estão disponíveis em acesso livre artigos publicados por 6 periódicos portugueses em ciências da saúde, nenhum deles dedicado exclusivamente ao campo da Saúde Pública ou especificamente à epidemiologia (anexo 2). Durante o período de 1996 a 2002 foram publicados seis números da **Revista de Epidemiologia,** que em verdade foram suplementos dos Arquivos de Medicina, que foi recentemente incluído no SciELO Portugal . Estes seis números encontram-se disponíveis em acesso livre no site da Associação Portuguesa de Epidemiologia(http:// ape.med.up.pt).

A **Revista Portuguesa de Saúde Pública** é editada pela Escola Nacional de Saúde Pública, da Universidade Nova de Lisboa, desde 1983. A publicação é semestral e a partir de 1999 conta também com a edição de números temáticos. A missão da revista é contribuir para a produção do conhecimento científico na área da saúde pública, promovendo a sua discussão e desenvolvimento, em Portugal e no mundo. Os textos são publicados em português com resumo em inglês. Estão disponíveis em acesso livre os números publicados entre 2000 e 2007 e paulatinamente os números anteriores estão sendo colocados em meio eletrônico. O acesso pela página da Universidade não é muito simples, entretanto, é possível obter acesso fácil através do Google Scholar.

**Indexação, Desempenho e Avaliação das Revistas**

A tabela 2 apresenta alguns dados cienciométricos para essas revistas na base SciELO.^5^ O número médio de citações por artigo publicado no período variou entre 0,52 para a revista História, Ciência, Saúde e 2,87 para a revista Physis. A vida média das citações foi mais homogênea com valores predominando na faixa dos três anos. Chama a atenção o valor de 7 anos para os artigos da revista Physis refletindo provavelmente o predomínio de ensaios teóricos característico dessa publicação. A Revista de Saúde Pública, embora publique predominantemente resultados de pesquisas empíricas também apresentou valor superior à média chegando a quase 5 anos.

Outros dados interessantes apresentados na Tabela 2 correspondem à porcentagem de citações recebidas provenientes de artigos publicados pela própria revista. Neste item, os dois periódicos indexados no JCR são os que apresentam maior proporção de autocitações recebidas, correspondendo a pouco mais da metade das citações. Chama a atenção a baixa taxa de auto citação da revista Physis. A proporção de autocitações concedidas é bastante menor, isto é, do conjunto de artigos citados pelos artigos publicados nesses periódicos uma parte pequena corresponde a citações de artigos publicados no mesmo periódico. Para todos os periódicos esse valor não ultrapassa os 10 por cento.

A figura 1 mostra a média de citações por artigo nos últimos 5 anos e em 2007, até o meio do ano. Os valores são bem superiores aos apresentados na Tabela 2 mostrando que a tendência dos últimos anos tem sido o aumento do número médio de citações por artigo publicado. Os valores observados em 2007 também superam a média do período para a maioria dos periódicos.

A Tabela 3 apresenta os periódicos nacionais e estrangeiros mais citados nos artigos publicados pelas revistas brasileiras de Saúde Pública e Epidemiologia na base SciELO. Os periódicos mais citados são aqueles com maior visibilidade em suas respectivas áreas demonstrando a familiaridade dos autores brasileiros com a produção científica mundial.^5^

A média de acessos mensais a textos completos está em torno de 7,5 milhões para os periódicos da coleção Brasil. Nos últimos 5 anos, três dos periódicos de Saúde Pública estiveram entre os mais visitados: Cadernos de Saúde Pública (11.049.923 acessos) em primeiro lugar, Revista de Saúde Pública (10,723,286 acessos) em segundo lugar e Ciência & Saúde Coletiva (4,148,533 acessos) em décimo primeiro lugar.^11^

Os Cadernos de Saúde Pública e a Revista de Saúde Pública estiveram também entre os periódicos mais citados na base: Cadernos de Saúde Pública recebeu 4079 citações (quarto lugar) e Revista de Saúde Pública, 4061 citações (quinto lugar).^11^

A Tabela 4 apresenta alguns dados relativos às estatísticas produzidas pela SciELO, relativas às oito revistas selecionadas. Embora cada uma delas tenha sido incluída na base em momentos distintos é possível verificar que todas elas apresentam grande freqüência de acessos com solicitação de texto completo. O número de acessos para o artigo mais acessado em cada uma delas variou entre 300 e 27900 solicitações. Um grande número de artigos recebeu mais de 500 acessos em cada uma delas e o número de artigos cujo texto completo foi solicitado é expressivo para todas , variando principalmente em função do tempo de inclusão na base.^5^

A proporção de artigos de pesquisadores brasileiros com colaboração de autores estrangeiros, publicados nos periódicos nacionais ainda é relativamente pequena. Entre 5 e 9% dos artigos trazem a colaboração de pesquisadores estrangeiros. Packer e Meneghini ^8^ analisaram os artigos com autores brasileiros que receberam mais de 100 citações na base de dados do Thomson Institute (ISI) no período de 1994-2003, em todas as áreas do conhecimento, e verificaram que para esses artigos a cooperação internacional chega a 84,3%.

Restringindo a análise aos artigos que receberam 250 citações ou mais a colaboração com autores estrangeiros aparece em 89,2% dos artigos.^9^ Os autores concluem que quanto mais restritivo é o critério de seleção dos artigos maior é o peso da cooperação internacional e da organização em redes. Embora os dados se refiram ao conjunto da produção científica brasileira, os autores informam que entre os artigos mais citados prevalecem as investigações médicas e epidemiológicas realizadas através de redes multinacionais.

**Internet and Open Access movement**

Professores, pesquisadores, alunos e funcionários de 188 instituições de ensino superior e de pesquisa em todo o País têm acesso imediato à uma parte importante da produção científica mundial atualizada através do Portal de periódicos CAPES mantido pelo Governo do Brasil a um custo anual da ordem de US$ 20 milhões.

O Portal de Periódicos CAPES ^10^ oferece acesso gratuito aos textos completos de artigos de mais de 11.419 revistas internacionais, nacionais e estrangeiras, e a mais de 90 bases de dados com resumos de documentos em todas as áreas do conhecimento. Inclui também uma seleção de importantes fontes de informação acadêmica com acesso gratuito na Internet. O uso do Portal é livre e gratuito para os usuários das instituições participantes, ou seja, todas as universidades públicas e as filantrópicas ou lucrativas cujos cursos de pós-graduação credenciados através da avaliação trienal de seus cursos de pós-graduação. O acesso é realizado a partir de qualquer terminal ligado à Internet localizado nas instituições ou por elas autorizadas.^10^

Todos os programas de pós-graduação, de pesquisa e de graduação do País ganham em qualidade, produtividade e competitividade com a utilização do Portal que está em permanente desenvolvimento. Entre janeiro e junho de 2007 o número de acessos com solicitação de texto completos no Portal foi de 7,4 milhões representando um custo médio por artigo de US$ 2,50.

**Discussão**

Apesar da língua inglesa ser cada vez mais considerada a língua franca nas ciências, os pesquisadores brasileiros, como sói acontecer com pesquisadores de outras nacionalidades cuja língua natal não é o inglês, normalmente enfrentam o dilema entre publicar seus trabalhos em inglês buscando aumentar a sua visibilidade na comunidade científica internacional ou publicar em sua língua materna buscando maior impacto social, ou seja, aproveitamento dos resultados da pesquisa para a solução de problemas concretos. Este dilema é bastante presente em áreas profissionais e aplicadas, como a Saúde Publica ^12^

Parker^11^ analisando a estrutura de indexação dos periódicos da América Latina e do Caribe em ciências de saúde observou que existem cerca de 1000 periódicos nessa área nos diferentes países. Destes, apenas 690 estão indexados na base LILACS, enquanto no portal SciELO esse número se reduz para 141. No Medline podem ser encontrados apenas 65 e no ISI-JCR somente 28. Deste modo fica bastante claro que a consulta a bases menos inclusivas produz o ocultamento de parte significante da produção. Tendo em vista que os critérios para indexação de periódicos na base LILACS seguem os mesmos padrões de exigência adotados pelo Medline, conclui-se que 90% da produção científica latino-americana e caribenha deixam de circular na comunidade científica internacional provavelmente em decorrência da barreira de linguagem.

Clark e Castro^13^ verificaram o impacto que a busca na base LILACS teria sobre a seleção de artigos para revisões sistemáticas publicadas nas cinco revistas médicas com maior fator de impacto. Em 70% dos casos analisados (62 revisões sistemáticas) a busca na LILACS identificou novos artigos passíveis de inclusão e que não haviam sido identificados pelos autores.

Outro aspecto importante que resulta da exclusão da literatura publicada em outras línguas que não o inglês é ressaltado por Freitas e colaboradores.^14^ Os autores mostram que a maioria dos ensaios clínicos publicados em inglês mostram resultados estatisticamente significantes enquanto menos da metade dos estudos publicados em outras línguas apresentam resultados positivos. A exclusão da literatura em português, espanhol e outras línguas pode induzir vieses importantes na realização de revisões sistemáticas.

Em revisão sistemática sobre morbidade e mortalidade materna realizaram-se buscas por artigos científicos em onze diferentes bases de dados, tendo-se observado que cerca de 20% dos artigos incluídos foram publicados em outras línguas que não o inglês ^15^. Os autores concluem que para esse tipo de revisão sistemática, baseada em estudos observacionais sobre a prevalência ou incidência de um evento de saúde, a busca de evidências em bases de dados regionais que indexam a produção de periódicos locais sem indexação no Medline é especialmente relevante.

É documentado o fato de que artigos publicados em outras línguas que não o inglês correm o risco de serem ignorados simplesmente por estarem escritos em línguas que não são acessíveis à comunidade científica internacional. Entretanto, "os autores geralmente pretendem atrair o interesse sobre seu trabalho tanto no âmbito nacional quanto internacional" como afirmam Meneghini e Packer.^12^ Segundo esses autores, os pesquisadores brasileiros publicam cerca de 50 mil artigos por ano dos quais 60% são divulgados em português. Destes, cerca de 18 mil são publicados em periódicos indexados por Thomson Scientific Web of Science database sendo 97,3% divulgados em inglês.

Barreto^1^ analisou a produção brasileira em epidemiologia indexada pelo MedLine entre 1984 e 2004. Usando uma combinação boleana de vários termos para selecionar artigos em epidemiologia, no início do período, 1984-1989, foram recuperados nessa base apenas 91 artigos de autores brasileiros correspondendo a 0,5% da produção mundial. Usando os mesmos critérios de busca, no final do período, 2000-2004, foram recuperados 1096 artigos correspondendo a 1,1% da produção internacional. Parte importante desses artigos - 38% - foi publicada em periódicos nacionais e os demais em inglês, principalmente em periódicos norte-americanos (23%) ou do Reino Unido (18%). Dos artigos publicados em periódicos nacionais 33% foram divulgados em inglês. Do total de artigos de epidemiologia produzidos por autores brasileiros em periódicos indexados pelo MedLine, 71% foram escritos em inglês.

Enquanto a maioria das revistas brasileiras de Epidemiologia e Saúde Pública aceitam artigos em espanhol e inglês, além do português, a Revista de Saúde Pública adotou recentemente a alternativa de seguir publicando seus artigos em inglês, português ou espanhol na forma impressa, mas traduzir todos os artigos para o inglês, na sua versão eletrônica. Com essa medida a Revista pretende manter seu compromisso social com a divulgação da produção científica nacional para os profissionais de saúde do país sem deixar de participar, na medida em que esta participação for possível, do debate científico internacional.

Em resumo, a pesquisa epidemiológica no Brasil consolida-se na mesma velocidade que outros campos científicos do país, buscando maior grau de internacionalização como é próprio do empreendimento científico em todas as áreas, porém, sem abandonar seu compromisso enquanto prática social afeita a ampliar a base dos conhecimentos sobre a situação e os determinantes de saúde da população brasileira visando a transformação da realidade e a conquista de melhores condições de vida e saúde para todos os brasileiros.

**Summary Box**

1. No Brasil, a produção científica em epidemiologia tem crescido substancialmente na última década

2. Parte considerável dessa produção está sendo publicada em inglês porém há um contingente não desprezível de resultados de pesquisa publicados exclusivamente em português

3. O Brasil, é o único pais de língua portuguesa que tem uma rede de revistas cientificas em Saúde publica/epidemiológica. No que se fere a língua em que os artigos são publicados estas revistas adotam a publicação em português, inglês, ou espanhol, embora a maioria dos artigos seja publicados em português.

4. A criação da base de open-acess SciELO auxiliou de maneira decisiva o fortalecimento dos periódicos brasileiros da área de Saúde Pública favorecendo uma maior comunicação da produção dos epidemiologistas brasileiros com os epidemiologistas do restante da América Latina, Espanha, Portugal e países africanos de língua portuguesa

5. A indexação dos principais periódicos brasileiros no MedLine tem possibilitado maior grau de internacionalização da pesquisa epidemiológica produzida no país

6. O compromisso social da comunidade de epidemiologistas brasileiros com a melhoria da saúde da população brasileira e o desenvolvimento e aprimoramento do Sistema Nacional de Saúde faz com que parte substancial da produção continue a ser veiculada em português.

**Referências Bibliográficas**

1. Barreto ML. **Crescimento e tendência da produção científica em epidemiologia no Brasil.** *Revista de Saúde Pública* 2006; 40 (N Esp):79-85.

2. CNPq. **Diretório de Grupos de Pesquisa. Plataforma Lattes**. [www.cnpq.br]. 2007

3. Guimarães R; Lourenço R: Cosac S**. A pesquisa em epidemiologia no Brasil**. *Revista de Saúde Pública* 2001;35:321-40.

4. **BVS.** [http://bvsmodelo.bvsalud.org/site/lilacs]. Acessado em 01/10/2007.

5. **SciELO**[www.scielo.org]. Acessado em 01/10/2007

6. SciELO 10 anos. **Apresentação.** 2007 [www.eventos.bvsalud.org]

7. IBICT. **Catálogo Coletivo Nacional**. [http://ccn.ibict.br]. Acesso em 01/10/2007.

8. Packer AL , Meneguini R. **Articles with authors affiliated to Brazilian institutions published from 1994 to 2003 with 100 or more citations: I - The weight of international collaboration and the role of the networks.** *Anais da Academia Brasileira de Ciências,* 2006; 78(4):841-853.

9. Meneguini R, Packer AL. **Articles with authors affiliated to Brazilian institutions published from 1994 to 2003 with 100 or more citations: II - Identification of thematic nuclei of excellence in Brazilian science.** *Anais da Academia Brasileira de Ciências*, 2006; 78(4):855-883.

10. CAPES. **Portal de Periódicos CAPES**. 2007 [www.capes.gob.br.]

11. Packer AL. **SciELO Brasil: estado de desenvolvimento**. 2006 [www.eventos. bvsalud.org]

12. Meneghini R & Packer A. **Is there science beyond English?** *European Molecular Biology Organization. EMBO Reports*, 2007; 8: 112-116.

13. Clark OAC e Castro AA. **Searching the Literatura Latino Americana e do Caribe em Ciências da Saúde (LILACS) database improves systematic reviews.** *International Journal of Epidemiology* 2002; 31:112-114.

14. Freitas AE, Herbert RD, Latimer J, Ferreira PH. **Searching the LILACS database for Portuguese and Spanish-language randomized trials in physiotherapy was difficult.** *Journal of Clinical Epidemiology*, 2005; 58:233-237.

15. Betrán AP, Say L. Gülmezoglu AM, Allen T, Hampson L. **Effectiveness of different databases in identifying studies for systematic reviews: experience from the WHO systematic review of maternal morbidity and mortality.** *BMC Medical Research Methodology,* 2005: 5(6):doi: 10.1186/1471-2288-5-6.

Tabela 1: Principais periódicos brasileiros de saúde pública e epidemiologia e suas bases de indexação, 2007.

| Título do periódico | Base bibliográfica | Ano inicial disponível em versão eletrônica | Língua de publicação | Periodicidade |
| --- | --- | --- | --- | --- |
| Cadernos de Saúde Coletiva | LILACS | 2002 | Português, inglês, espanhol ou francês | trimestral |
| Cadernos de Saúde Pública | JCR , Medline, SCOPUS, LILACS, Scielo Saúde Pública, DOAJ | 1985 | Português, espanhol ou inglês | mensal |
| Ciência & Saúde Coletiva | Medline, SCOPUS, LILACS, Scielo Saúde Pública, DOAJ. | 1998 | Português, espanhol ou inglês | bimestral |
| Epidemiologia e Serviços de saúde | LILACS, Acesso eletrônico pela BVS* | 2003 | português | trimestral |
| História, Ciência, Saúde, Manguinhos. | Medline, LILACS, SCPOPUS Scielo coleção Brasil, DOAJ. | 1994 | Português, espanhol inglês ou francês | trimestral |
| Interface | Scielo coleção Brasil, LILACS, SCOPUS, DOAJ. | 2005 | Português, espanhol ou inglês | semestral |
| Physis | Scielo coleção Brasil, LILACS, SCOPUS DOAJ. | 2004 | Português, espanhol ou inglês | semestral |
| Revista Baiana de Saúde Pública | LILACS | 2004 | português | semestral |
| Revista Brasileira de Epidemiologia | LILACS, SCOPUS Scielo Saúde Pública, DOAJ. | 1998 | Português, espanhol ou inglês | trimestral |
| Revista Brasileira de Saúde Materno Infantil | LILACS, SCOPUS Scielo coleção Brasil, DOAJ. | 2002 | Português, espanhol ou inglês | trimestral |
| Revista Brasileira de Vigilância Sanitária |  | 2006 | Português, inglês ou espanhol | trimestral |
| Revista Brasileira em Promoção da Saúde | Latindex e DOAJ | 2003 | Português, inglês ou espanhol | trimestral |
| Revista da Escola Mineira de Saúde Pública |  | 2007 | Português, inglês ou espanhol | bimestral |
| Revista de Atenção Primária em Saúde |  | 2003 | português | semestral |
| Revista de Saúde Pública | JCR, Medline, LILACS, SCOPUS, Scielo Saúde Pública, DOAJ. | 1967 | Português, espanhol ou inglês* | bimestral |
| Saúde e Sociedade** | LILACS | 1992 | Português, inglês, espanhol ou francês | quadrimestral |
| Saúde em Debate | LILACS | Sem acesso eletrônico | português |  |

BVS- Biblioteca Virtual em Saúde (http://scielo.iec.pa.gov)

Tabela 2: Indicadores cienciométricos das revistas brasileiras de Saúde Pública e Epidemiologia na base SciELO, 1999 a 2007

| Periódico | Citações por artigo | Vida média  (a) | Citações auto recebidas (%) | Citações auto concedidas (%) | Colaboradores estrangeiros (%) |
| --- | --- | --- | --- | --- | --- |
| Cadernos de Saúde Pública | 1,78 | 3,92 | **58,0** | 6,6 | 6,2 |
| Ciência & Saúde Coletiva | 1,07 | 3,38 | 32,9 | 2,8 |  |
| História, Ciência, Saúde, Manguinhos | **0,52** | 3,49 | 52,3 | 2,9 | 5,2 |
| Interface | 0,38 | 3,29 | 28,9 | 4,7 | 6,1 |
| Physis | **2,87** | **7,04** | **3,7** | 3,2 | 5,1 |
| Revista Brasileira de Epidemiologia | 0,77 | 3,33 | 19,2 | **0,0** | **3,7** |
| Revista Brasileira de Saúde Materno Infantil | 0,67 | **2,61** | 37,5 | 1,3 | 8,2 |
| Revista de Saúde Pública | 2,09 | 4,95 | 52,9 | **8,7** | **9,2** |

Figura 1:

Tabela 3: Periódicos nacionais e estrangeiros mais citados em artigos publicados nos periódicos nacionais de Saúde Pública e epidemiologia, base SciELO, 1999-2007

| Periódico | Nº citações | % citações concedidas |
| --- | --- | --- |
| Revista de Saúde Pública | 5600 | 30,0 |
| Cadernos de Saúde Pública | 4453 | 23,9 |
| Lancet | 1396 | 7,5 |
| American Journal of Epidemiology | 1119 | 6,0 |
| Memórias do Instituto Oswaldo Cruz | 1056 | 5,7 |
| British Medical Journal | 975 | 5,2 |
| American Journal of Public Health | 689 | 3,7 |
| American Journal of Clinical Nutrition | 586 | 3,1 |
| Revista da Sociedade Brasileira de Medicina Tropical | 521 | 2,8 |
| Japanese Journal of Medical Science- Social Medicine | 484 | 2,6 |
| JAMA | 451 | 2,4 |
| Revista do Instituto de Medicina Tropical | 392 | 2,1 |
| American Journal of Tropical Medicine and Hygiene | 369 | 2,0 |
| Boletin de la Oficina Sanitaria Panamericana | 298 | 1,6 |
| Ciencia & Saúde Coletiva | 273 | 1,5 |
| TOTAL | 18662 | 100,0 |

Tabela 4: Estatísticas de acesso a artigos (solicitação de texto completo) para as revistas de Saúde Pública e Epidemiologia na base SciELO, 1999 a 2007.

| Revista | Nº de acessos para o artigo mais acessado | Nº de artigos com mais de 500 acessos | Inclusão na base | Nº artigos solicitados |
| --- | --- | --- | --- | --- |
| Cadernos de Saúde Pública | 20981 | 1746 | 1999 | 3905460 |
| Ciência & Saúde Coletiva | 561 | 3 | 2002 | 26718 |
| História, Ciência, Saúde, Manguinhos | 22963 | 690 |  |  |
| Interface | 9588 | 111 | 2005 | 253240 |
| Physis | 17745 | 71 | 2005 | 147659 |
| Revista Brasileira de Epidemiologia | 300 | 69 | 2005 | 10054 |
| Revista Brasileira de Saúde Materno Infantil | 27901 | 254 | 2003 | 805091 |
| Revista de Saúde Pública | 21667 | 1562 | 1999 | 3302642 |

ANEXO 1

Revista brasileiras da área de Ciências da Saúde na base SciELO, não classificadas como de Saúde Pública ou Epidemiologia

ANEXO II

Total de Revistas portuguesas da área de Ciências da Saúde na Base SciELO
