# Supplementary material for: Public health and epidemiology journals published in Brazil and other Portuguese speaking countries
Source: Emerg Themes Epidemiol. 2008 Sep 30;5:18. doi: 10.1186/1742-7622-5-18 (PMC2572600; doi:10.1186/1742-7622-5-18)
Supplement: Additional file 2 — Abstract in simplified Chinese [file 1742-7622-5-18-S2.pdf]

Simplifed Chinese / 简体中文

分析透视

在巴西和其他葡萄牙语国家出版的公共卫生及流行病学期刊

作者：Mauricio L. Barreto, Rita Barradas Barata

摘要

众所周知以非英语语文写作的文章有一个大风险，就是没有人注意那篇文章，因为国际科学社群没有掌握到这些语文。这篇文章的目的是要促进人们使用葡萄牙语国家的公共卫生及流行病学文献。这些文献特别集中在巴西，另有一些来自葡萄牙，但却没有来自其他葡萄牙语国家的。这些文献以葡萄牙文为主，但也有以英文或西班牙文写作的。在这篇文章里，我们描述了以葡萄牙文出版的公共卫生及流行病学期刊和为它们编纂目录的文献目录数据库，以及读取这些期刊的方法。通过目录数据库里的直接网上连结，人们可以免费读取绝大部份的期刊的文章。我们也讨论了葡萄牙语科研成果对流行病学作为一门科学学科及作为一门公共卫生实践的基础学科的发展的重要性。这些文献的边缘化牵涉到我们能否对全球健康问题及它们的决定因子建立一个更平衡的认识与了解。

（中文摘要由冯俊熙翻译）
