# Supplementary material for: Public health and epidemiology journals published in Brazil and other Portuguese speaking countries
Source: Emerg Themes Epidemiol. 2008 Sep 30;5:18. doi: 10.1186/1742-7622-5-18 (PMC2572600; doi:10.1186/1742-7622-5-18)
Supplement: Additional file 3 — Abstract in traditional Chinese. [file 1742-7622-5-18-S3.pdf]

Traditional Chinese / 繁體中文

分析透視

在巴西和其他葡萄牙語國家出版的公共衛生及流行病學期刊

作者：Mauricio L. Barreto, Rita Barradas Barata

摘要

眾所周知以非英語語文寫作的文章有一個大風險，就是沒有人注意那篇文章，因為國際科學社群沒有掌握到這些語文。這篇文章的目的是要促進人們使用葡萄牙語國家的公共衛生及流行病學文獻。這些文獻特別集中在巴西，另有一些來自葡萄牙，但卻沒有來自其他葡萄牙語國家的。這些文獻以葡萄牙文為主，但也有以英文或西班牙文寫作的。在這篇文章裡，我們描述了以葡萄牙文出版的公共衛生及流行病學期刊和為它們編纂目錄的文獻目錄數據庫，以及讀取這些期刊的方法。通過目錄數據庫裡的直接網上連結，人們可以免費讀取絕大部份的期刊的文章。我們也討論了葡萄牙語科研成果對流行病學作為一門科學學科及作為一門公共衛生實踐的基礎學科的發展的重要性。這些文獻的邊緣化牽涉到我們能否對全球健康問題及它們的決定因子建立一個更平衡的認識與了解。

（中文摘要由馮儁熙翻譯）
