# Supplementary material for: Public health and epidemiology journals published in Brazil and other Portuguese speaking countries
Source: Emerg Themes Epidemiol. 2008 Sep 30;5:18. doi: 10.1186/1742-7622-5-18 (PMC2572600; doi:10.1186/1742-7622-5-18)
Supplement: Additional file 4 — Abstract in French. [file 1742-7622-5-18-S4.pdf]

French / Français

Perspectives analytiques

Les revues de santé publique et d’épidémiologie publiées au Brésil et autres pays de langue portugaise

Auteurs: Mauricio L. Barreto, Rita Barradas Barata

Résumé

Il est bien connu que les articles écrits dans une langue autre que l’anglais risquent fortement d’être ignorés simplement parce que ces langues ne sont pas accessibles à la communauté scientifique mondiale. L’objectif de cet article est de faciliter l’accès à la littérature en santé publique et épidémiologie disponible dans les pays de langue portugaise. La littérature se concentre particulièrement au Brésil, avec quelques contributions du Portugal, et aucune provenant des autres pays de langue portugaise. Elle est essentiellement écrite en portugais, mais aussi en d’autres langues telles l’anglais ou l’espagnol. Dans cet article, nous décrivons les revues publiant la littérature en santé publique et épidémiologie en portugais, les bases de données utilisées pour leur indexation, et comment l’on peut accéder à ces revues. La plupart des revues sont librement accessibles à travers des liens directs aux articles dans les bases de données d’indexation. Nous discutons également de l’importance de la publication de la recherche scientifique en portugais pour le développement de l’épidémiologie comme discipline scientifique et comme discipline

de base pour la pratique de la santé publique. La marginalisation de ces publications a des conséquences en ce qui concerne la connaissance et la compréhension plus équilibrées des problèmes de santé et leurs déterminants à l’échelle mondiale.

Traduit de l’anglais par Philip Harding-Esch
