# Supplementary material for: Public health and epidemiology journals published in Brazil and other Portuguese speaking countries
Source: Emerg Themes Epidemiol. 2008 Sep 30;5:18. doi: 10.1186/1742-7622-5-18 (PMC2572600; doi:10.1186/1742-7622-5-18)
Supplement: Additional file 5 — Abstract in Spanish [file 1742-7622-5-18-S5.pdf]

Spanish / Español

Perspectivas analíticas

Revistas de salud pública y epidemiología publicadas en Brasil y otros países de habla portuguesa.

Autores: Mauricio L. Barreto, Rita Barradas Barata.

Resumen

Es reconocido que los artículos escritos en idiomas otros que el inglés corren el riesgo de ser ignorados porque estos idiomas no son accesibles a la comunidad científica internacional. Este artículo tiene como objetivo facilitar el acceso a la literatura en salud pública y epidemiología disponible en países de lengua portuguesa. La literatura proviene mayoritariamente de Brasil, con algunas contribuciones de Portugal pero ninguna de otros países de habla portuguesa. Los artículos son escritos predominantemente en portugués, pero también en otros idiomas como el inglés o el español. En este artículo describimos las revistas que publican literatura en salud pública y epidemiología en portugués, las bases de datos bibliográficas que las indexan y cómo se puede acceder a estas revistas. La mayoría de las revistas son accesibles gratuitamente a través de un vínculo al artículo listado en las bases de datos. También discutimos la importancia de la producción científica en portugués para el desarrollo de la epidemiología como disciplina científica y como disciplina de base para la práctica en salud pública. La marginalización de estas publicaciones tiene

implicaciones para un conocimiento y entendimiento más equilibrados de los problemas de salud y de sus determinantes a nivel mundial.

(Traducido por Annick Bórquez)
